# He Tamariki Kokoti Tau-Tackling Preterm: a data-linkage methodology to explore the clinical care pathway in preterm deliveries

**DOI:** 10.1186/s12913-018-3179-6

**Published:** 2018-05-21

**Authors:** Sara Filoche, Fiona Cram, Angela Beard, Dalice Sim, Stacie Geller, Liza Edmonds, Bridget Robson, Beverley Lawton

**Affiliations:** 1 Department of Obstetrics and Gynaecology and Department of Pathology and Molecular Medicine, University of Otago, Wellington, New Zealand; 2 Katoa Ltd, Auckland, New Zealand; 3 Christchurch Obstetric Associates, Christchurch, New Zealand; 4 Dean’s Department, University of Otago, Wellington, New Zealand; 5 Department of Obstetrics and Gynecology, University of Illinois, Chicago, USA; 6 Women’s and Children’s Health, Paediatrics & Child Health, Dunedin School of Medicine, Health Sciences and Southern District Health Board, Dunedin, New Zealand; 7 Te Rōpū Rangahau Hauora a Eru Pōmare, University of Otago, Wellington, New Zealand; 8 Centre for Women’s Health Research-Te Tātai Hauora O Hine, Faculty of Health, Victoria University of Wellington, Wellington, New Zealand

**Keywords:** Indigenous health, Preterm delivery, Data linking, Equity, Clinical care pathway, Kaupapa Māori, Maternity care services, Health disparities

## Abstract

**Background:**

Significant health inequities exist around maternal and infant health for Māori, the indigenous people of Aotearoa New Zealand – and in particular around a premature (preterm) delivery. Māori babies are more likely to be born preterm (8.1%, compared to an overall rate of 7.4%) and they are more likely to have a preterm death. An essential part of redressing these disparities is to examine the clinical care pathway and outcomes associated with preterm deliveries. This paper describes a protocol utilising national and local health collections to enable such a study.

**Design:**

This is a retrospective cohort study comprising 5 years data pertaining to preterm deliveries from 2010 to 2014. These data are generated from linked national administrative and local health information collections to explore a range of neonatal outcomes and infant mortality in relation to the antenatal care pathway and known risk factors for preterm delivery. This study is being conducted within a Kaupapa Māori paradigm that dismisses victim blaming and seeks to intervene at structural levels to improve the health and wellbeing of Māori whānau (family).

**Significance of the study:**

Our data-linkage methodology optimises the utility of New Zealand health collections to address a significant health issue. Our findings will fill the information gaps around the burden of preterm delivery by quantifying the incidence of preterm delivery and adverse neonatal and infant outcomes in Aotearoa New Zealand. It will explore access to evidenced based care including use of steroids before birth, and appropriate place of delivery. The results from this study will inform maternity care services to improve management of preterm deliveries – both locally and internationally. This in turn will improve the preterm sequela by reducing the long-term health burden and health inequities.

## Background

Premature deliveries (preterm) represent a global health burden [1] for which there are significant disparities by ethnicity [2, 3]. In Aotearoa New Zealand such health inequities exist between Māori, the indigenous people of Aotearoa New Zealand, and Pākehā (New Zealand European) [4–7].

This paper describes the protocol for a retrospective cohort study that links local hospital, community laboratories and national health information datasets to explore the preterm delivery clinical care pathway, delivery and neonatal outcomes and infant mortality (≤ 1 yr. of age) of Māori and Pākehā (New Zealand European) women and their babies. Building on an approach we have previously described, [8, 9] we have further developed this methodology to enable an in-depth exploration of key elements of antenatal care such as the administration of corticosteroids in relation to neonatal and infant outcomes.

### Preterm deliveries in Aotearoa New Zealand

A premature delivery (preterm) can potentially result in adverse outcomes such as death, brain haemorrhage, infection, chronic lung disease and long-term growth impairment and harm^40^ including cerebral palsy, cognitive, visual and learning impairments [10–14]. Māori whānau (families) suffer a greater burden of this preterm harm and death than Pākehā. We have known of these disparities since Mantel’s early work in the 1990s, with his findings repeatedly confirmed by others including Robson [7], Craig [15] and the Perinatal and Maternal Mortality Review Committee (PMMRC) [4]. Māori babies are most likely to be born preterm (8.1%, compared to an overall rate of 7.4%) and they are more likely to have a preterm death [4]. There are approximately 1200 Māori preterm births annually. Preterm is the second commonest cause of peri-natal death in Aotearoa accounting for 20% of deaths [4]. Māori have over twice the rate of preterm death compared to Pākehā (359 Māori babies died from 2007 to 2013 compared to 319 Pākehā) [4]. An essential part of redressing these disparities is to examine the clinical care pathway associated with preterm deliveries. This paper describes a protocol utilising local and national administrative health collections to enable such a study.

## Design

This is a retrospective cohort study comprising 5 years data pertaining to preterm deliveries from 2010 to 2014. These data are generated from linked national administrative and local health information collections to explore a range of neonatal outcomes and infant mortality in relation to the antenatal care pathway and known risk factors for preterm delivery such as the lack of antenatal corticosteroids prior to delivery or delivery at the appropriate care centre [16, 17].

This study is being conducted within a Kaupapa Māori paradigm that dismisses victim blaming and seeks to intervene at structural levels to improve the health and wellbeing of Māori whānau. It seeks to identify where resources may be best allocated to improve Māori health and wellbeing. Kaupapa Māori Research is about ensuring good health outcomes for Māori and frames our approach [18–20].

### Outcome measures

#### Primary outcomes

1. To compare for Māori and Pākehā the proportion for infants born preterm having at least one of the following outcomes:

- Death, up to 1 year post birth
- Brain injury (defined as periventricular and/or cerebellum haemorrhage and/or Periventricular Leucomalacia (PVL))
- Chronic lung disease of infancy (Bronchopulmonary BPD subtype)
- Oxygen and/or tube feeding on discharge
- Length of stay (LOS) (until first discharge home) at hospital > 4 months

2. To compare for Māori and Pākehā the national rate of infants born preterm with at least one of the following outcomes: death, small for gestational age and chronic lung disease of infancy.

#### Secondary outcomes

To compare for Māori and Pākehā women the proportion of:

1. Women delivering preterm who receive full-course antenatal corticosteroid administration
2. The percentage of infants born preterm
3. Preterm infants not born at an appropriate level hospital (such as a tertiary hospital).
4. Preterm infants with retinopathy of prematurity and or deafness
5. Women who have a preterm delivery who receive appropriate antenatal screening

### Study population

The study population includes pregnant women and their preterm babies born between 2010 and 2014 in Aotearoa New Zealand. For Primary Outcome 1, the study population includes pregnant women and their preterm infants born in 5 different district health board (DHBs) regions.

#### Inclusion criteria

All infants born to women between 24^0^ and 36^6^ completed week’s gestation between January 1st 2010 and 31^st^ December 2014.

#### Exclusion criteria

Women not domiciled in the respective DHBs at the time of delivery (Primary Outcome 1 only). Infants less than 24 and greater than 36 weeks gestation will be excluded.

### Data sources

#### National health and clinical information collections in Aotearoa New Zealand

The data for our study is sourced from routine health information collections at both national and local levels (Table 1). In Aotearoa New Zealand, the Ministry of Health is responsible for the oversight and funding of New Zealand’s 20 DHBs. Select clinical information is reported by DHBs to the Ministry of Health, and is collated into national datasets with operational responsibility by the Client Insights and Analytics group. Among the wide range of health related registries and datasets held in New Zealand are: National Minimum Data Set (NMDS, covering hospital discharges), Mortality Collection, and National Maternity Collection (MAT) [21].

**Table 1:** Local and national data sources and examples of variables collected

Dataset	Type of information	Examples of variables extracted
National health index	Sociodemographic	NHI number, area deprivation, ethnicity
Mortality collection	Mortality	Ethnicity, date of death, gestational age at termination of pregnancy, birth-weight, diagnostic codes on cause of death, sudden and unexpected death indicator
National maternity collection	Pregnancy and delivery information	Maternal ethnicity, maternal height and weight at time of booking with a maternity care provider, maternal smoking status at time of booking with maternity care provider, plurality, parity, mode of delivery, Apgar at 5 min, birth weight, gestational age at delivery
National minimum dataset	Hospital events	Ethnicity, maternal and/or infant hospital admissions (public) and discharge dates, length of stay, diagnostic (ICD-10) codes
Australia New Zealand Neonatal network	Neonatal intensive care	Ethnicity, corticosteroid administration before delivery, length of stay, neonatal surgery, corrected gestational age on discharge, oxygen on discharge, enteral tube feeding on discharge (e.g. nasogastric tube)
Local hospital (DHB specific)	Pregnancy and delivery events	Maternal and paternal ethnicity, obstetric history, plurality, parity, date of admission, date of delivery
Community laboratory	Screening and diagnostics	Antenatal booking screening (e.g. blood count), gestational diabetes screening, sexually transmitted infection (note: only if screened and not results)

In addition, there is an Australia New Zealand Neonatal Network (ANZNN) collection which is a collaboration of every neonatal intensive care unit in the two countries, established in 1994 [22]. The inclusion criteria for registration in this dataset are babies admitted to a neonatal unit who meet one or more of the following:

- born at less than 32 weeks gestation, or
- weighed less than 1500 g at birth, or
- received assisted ventilation (mechanical ventilation) including intermittent positive pressure ventilation (IPPV) or continuous positive airways pressure (CPAP) or high flow nasal cannulae for four or more consecutive hours, or died while receiving mechanical ventilation prior to 4 hours of age, or
- received major surgery (surgery that involved opening a body cavity), or
- received therapeutic hypothermia

### Local health and clinical information collections in Aotearoa New Zealand

Clinical information that is not reported to the Ministry of Health (so does not appear in these national collections) is held locally within independent information systems maintained within each DHB: for example the Perinatal Information Management System (PIMS), which collects information on perinatal events (Table 1).

In Aotearoa community laboratories offer a range of services; taken as indicator/proxy of receiving appropriate antenatal care (as these tests are usually carried out when the woman registers with her maternity care provider and during pregnancy) and relevant to this study include antenatal blood tests such as blood count and gestational diabetes screening.

### Data collection and matching

The variables retrieved from each collection (as described in the associated data dictionaries) were discussed in consultation with the research team and were informed by the Client Insights and Analytics group who were able to provide information pertaining to the quality of data (e.g. missing data). Risk factor variables (confounders for analysis) for preterm delivery were also discussed in-line with available data and where possible were collected (e.g. maternal smoking status) or constructed (e.g. inter-timing pregnancy and previous preterm delivery).

**Fig. 1:**
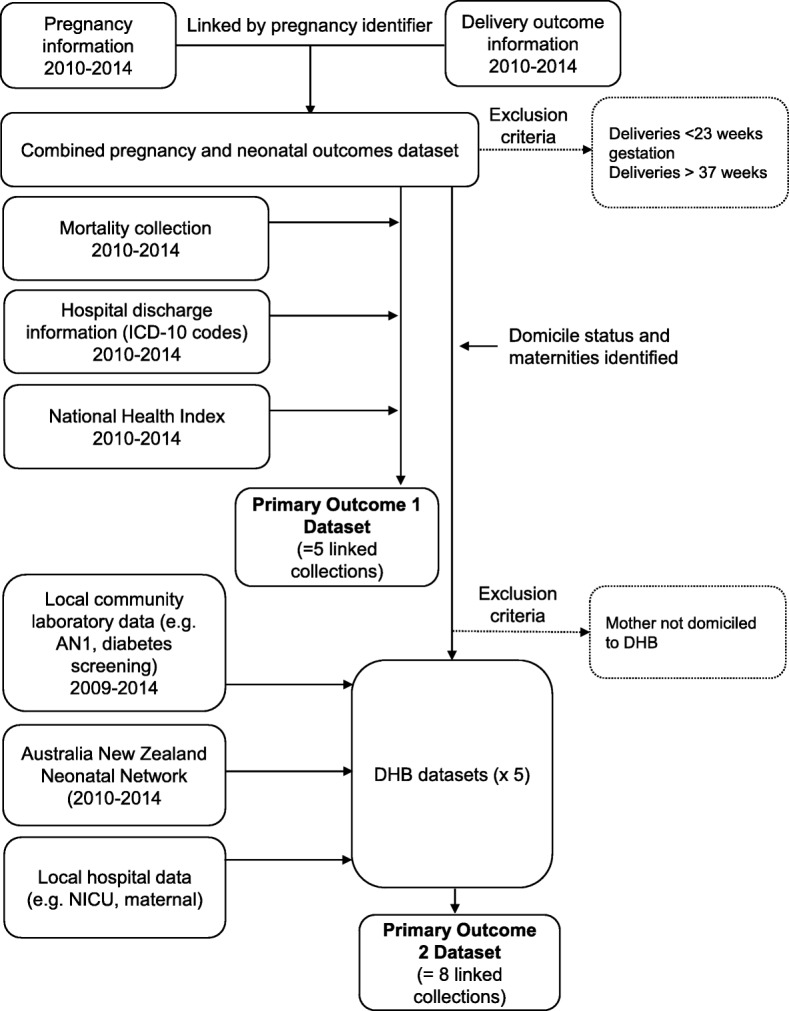
Overview of study protocol outlining the main data collation steps and exclusion criteria for each Primary Outcome AN1 (Antenatal booking). NICU (Neonatal Intensive Care Unit)

For Primary Outcome 2, two datasets, pregnancy information and fetal-neonatal outcome information, are joined using the unique pregnancy identifier as outlined in Fig. 1 (and cross referenced with hospitalisation discharge information as a quality checking step). The pregnancy identifier is unique for each pregnancy event, and enables clear delineation of each pregnancy and fetal-neonatal outcome information. For example, if a pregnancy is multiparous e.g. twin, in the pregnancy dataset the identifier would appear once and twice in the fetal-neonatal outcome dataset. Using a combination of linking approaches with infant and maternal NHI numbers, the pregnancy and fetal-neonatal outcome dataset are linked to the hospitalisation discharge information (which contains ICD-10 codes) and mortality collection.

Based on domicile status from the combined pregnancy-fetal-neonatal dataset we have identified relevant pregnancies for Primary Outcome 1 (Fig. 1). Using maternal NHIs the community laboratory and hospital data are arranged so that each line in the dataset represents pregnancy information with fetal-neonatal outcome and also antenatal care-pathway elements of interest for that pregnancy. For example, whether the mother received gestational diabetes screening (which comprises a constructed variable flagged as yes or no; and is calculated based on whether screening occurred at the appropriate time according to guidelines at the time and at any time during pregnancy). In order to capture the antenatal screening data for deliveries in 2010, data from community laboratories were extracted from 2009.

Each collection is systematically explored for missing variables, and “cleaned” to remove any duplicate entries before any dataset linking or any variables are constructed e.g. number of repeat pregnancies and inter-timing pregnancy.

No personally identifiable data (i.e. names, addresses) have been or will be extracted from any of the data sources.

### Statistical analysis

In Aotearoa New Zealand there are approximately 60,000 live births per annum which for 5 years data would equate to 300, 000 delivery events. Based on a national average of preterm deliveries of 7.5% [4] our estimated population will comprise approximately 21, 000 preterm delivery events.

Sample size estimates are presented for Primary Outcome 1. To estimate the number of Māori and Pākehā babies born preterm per year between 2010 and 2014 we used annual maternity data on births for each of the 5 DHBs, and based the proportion of births that were preterm for Māori and Pākehā on a tertiary level DHB (approximately 11.7%, 65 /559 cases of preterm birth per annum for Māori and 8.1%, 270/3344 for Pākehā mothers). Using these assumptions, there are approximately 654 Maori preterm births and 1236 Pākehā preterm births per year across the 5 DHBs. Working from a baseline assumption that the preterm infants of Pākehā women will have an 8% rate of poor outcomes (ANZNN overall rate for chronic lung disease in infants at 36 weeks) [23, 24] with 1 year’s worth of data, this study will have 80% power (using an alpha of 0.05) to detect a significantly enhanced risk if poor outcomes are 11.7% or higher among Māori infants. If 2 years’ worth of data is used (1308 Māori preterm births and 2470 Pākehā preterm births), the study will have 80% power (using an alpha of 0.05) to detect a significantly higher risk if poor outcomes are 10.5% or higher in Māori infants.

For Primary Outcome 1 multivariable Poisson regression models will compare the rates of poor outcomes between preterm infants of Māori and Pākehā mothers, after adjusting for potential personal and care-related confounders [25–27] e.g. maternal age, rapid repeat pregnancies, antenatal screening, parity, socioeconomic status and smoking, and to account for the degree to which ethnic disparities in infant health outcomes are mediated by these factors. Descriptions of rates, rate ratios and respective 95% confidence intervals will be reported.

## Discussion

This is a multi-disciplinary collaborative study that aims to bring about positive changes in antenatal care for women at risk of having preterm delivery, and consequently for their infants.

Our data-linkage methodology optimises the utility of New Zealand health collections to address a significant health issue [1, 28, 29]. Our findings will fill the information gaps around the burden of preterm delivery by quantifying incidence of preterm delivery and adverse neonatal and infant outcomes in Aotearoa New Zealand [30]. It will explore access to evidence based care including use of steroids before birth, and appropriate place of delivery. The results from this study will inform maternity care services, both nationally and internationally. This study aligns with the New Zealand government goals to deliver quality health services, improve system performance, and reduce disparities in health. A complimentary qualitative study exploring the experiences of Māori whānau whose newborn has been admitted to neonatal intensive care because of a preterm delivery is also being carried out alongside this investigation (He Tamariki Kokoti Tau-Addressing Preterm).

The described study utilises both local and national level data collections and through a process of linking by unique identifiers is able to quantitatively explore and analyse the preterm care pathway and its outcomes. We are informed both by clinical expertise in the area of obstetrics and neonatalogy – both from the research team and local investigators. We are also informed by our kaumātua (Māori elders). The analysis for this study is still underway. It is expected that this in-depth investigation from a mother to infant perspective will provide a greater understanding of the clinical care pathway in preterm deliveries, which will inform local national health care policy and procedures around such maternities. As such maternity care providers will be able to identify high risk patients, intervene earlier and provide more appropriate management. This will in turn reduce the health burden of being “born too soon” [29] and also redress the health inequity associated with preterm deliveries, both within Aotearoa New Zealand and globally.

## Abbreviations

ANZNN: Australia New Zealand Neonatal Network
CPAP: Continuous positive airways pressure
DHB: District health board
IPPV: Intermittent positive pressure ventilation
LOS: Length of stay
MAT: National maternity collection
NICU: Neonatal intensive care unit
NMDS: National minimum data set
PIMS: Perinatal information management system
PMMRC: Perinatal and maternal mortality review committee
PVL: Periventricular Leucomalacia
